# Analysis of Biological Screening Compounds with Single- or Multi-Target Activity via Diagnostic Machine Learning

**DOI:** 10.3390/biom10121605

**Published:** 2020-11-27

**Authors:** Christian Feldmann, Dimitar Yonchev, Jürgen Bajorath

**Affiliations:** LIMES Program Unit Chemical Biology and Medicinal Chemistry, Department of Life Science Informatics, B-IT, Rheinische Friedrich-Wilhelms-Universität, Endenicher Allee 19c, D-53115 Bonn, Germany; cfeldmann@bit.uni-bonn.de (C.F.); yonchev@bit.uni-bonn.de (D.Y.)

**Keywords:** screening compounds, biological assays, single- vs. multi-target activity, polypharmacology, chemical biology, large-scale data analysis, diagnostic machine learning, structural relationships

## Abstract

Predicting compounds with single- and multi-target activity and exploring origins of compound specificity and promiscuity is of high interest for chemical biology and drug discovery. We present a large-scale analysis of compound promiscuity including two major components. First, high-confidence datasets of compounds with multi- and corresponding single-target activity were extracted from biological screening data. Positive and negative assay results were taken into account and data completeness was ensured. Second, these datasets were investigated using diagnostic machine learning to systematically distinguish between compounds with multi- and single-target activity. Models built on the basis of chemical structure consistently produced meaningful predictions. These findings provided evidence for the presence of structural features differentiating promiscuous and non-promiscuous compounds. Machine learning under varying conditions using modified datasets revealed a strong influence of nearest neighbor relationship on the predictions. Many multi-target compounds were found to be more similar to other multi-target compounds than single-target compounds and vice versa, which resulted in consistently accurate predictions. The results of our study confirm the presence of structural relationships that differentiate promiscuous and non-promiscuous compounds.

## 1. Introduction

The term compound promiscuity is often used with opposing connotations including the presence of non-specific, artificial, or otherwise undesired binding effects on the one hand, and the ability to engage in specific interactions with multiple targets on the other [1]. The latter ability has been rationalized as a form of “selectively nonselective” interactions [2], at least for closely related targets such as protein kinases [2]. However, multi-target activity of small molecules is not limited to targets from the same family, but extends to distantly or virtually unrelated targets [3]. Promiscuous compounds with specific multi-target activity are essential for eliciting polypharmacological effects in vivo, which are often of critical importance for the treatment of multi-factorial diseases [4,5,6,7,8]. By contrast, compounds with multi-target activity are problematic for chemical biology and target validation because small molecule probes for interrogating biological functions of individual targets should be as specific as possible [9,10]. 

Given the dual role of multi-target activity for polypharmacology-driven drug discovery, on the one hand, and chemical biology, on the other, one would like to better understand molecular characteristics that distinguish non-promiscuous and promiscuous compounds. However, rationalizing multi-target activity at the molecular level of detail is still in its early stages. While molecular property analysis has indicated that high hydrophobicity or the presence of positively-charged groups are often implicated in undesired target engagement and ensuing side effects [11,12] our current knowledge of structural characteristics that might be responsible for well-defined multi-target activity is limited. Insights into the molecular origins of promiscuity would also aid in the design of small molecules with pre-defined multi-target activities [13,14,15,16,17]. Designing such ligands is not only of high interest for polypharmacology, but also relevant for the concomitant use of chemical probes with defined dual-target activity and corresponding single-target activity, which enables the detection of synergistic functional effects. Dual-target ligand design frequently attempts combining pharmacophore elements from separate sets of compounds with known activity against two targets [13,14]. In addition to generating such hybrid compounds, scaffolds shared by compounds having different activity can be considered as templates for multi-target ligand design [18]. 

Promiscuity analysis substantially benefits from computational support. For example, ligand-based computational approaches have been used to predict new targets of known active compounds [19,20]. These approaches infer compound-target relationships from a calculated similarity of known ligands. The identification of new targets for pharmaceutically relevant compounds originally thought to be specific renders these compounds potential candidates for polypharmacology and often also explains undesired side effects observed for clinical compounds. Computational target identification methods are also applied for repurposing existing therapeutic agents [21]. Furthermore, these approaches are relevant for chemical biology because they can aid in evaluating and prioritizing chemical probes. 

Different types of computational predictions have focused on systematically distinguishing between promiscuous and non-promiscuous compounds using machine learning (ML) [22,23]. These studies have shown that promiscuous and non-promiscuous compounds from medicinal chemistry sources were predictable with reasonable accuracy on the basis of chemical structure, indicating the presence of structural patterns that differentiate compounds with single- and multi-target activity. Successful predictions were obtained for promiscuous compounds active against related or unrelated targets, including proteins from functionally distinct classes [23]. These studies revealed that nearest neighbor (NN) relationships between promiscuous or non-promiscuous compounds strongly contributed to the predictions. These relationships resulted from the preferential formation of series of structural analogs by many promiscuous and non-promiscuous compounds, respectively. The presence of distinct analog series comprising promiscuous and non-promiscuous compounds rationalized the predictions including the strong influence of NN effects [23].

For compounds from the medicinal chemistry and patent literature, experimental test frequencies are generally unknown. For promiscuity analysis, this causes a potential bias in cases where assay frequencies of related compounds significantly differ. For example, if a given compound was tested only against a few targets but a structural analog against many different ones, the latter compound would more likely to display multi-target activity than the former for statistical reasons. Hence, the availability of test frequency information lends further credence to compound promiscuity assessment and predictions. This information can be obtained for compounds from biological screening campaigns, which has been a major motivation for our current investigation. Herein, we present newly designed high-confidence compound sets for promiscuity analysis extracted from biological screening data and report diagnostic ML studies under varying conditions to distinguish between multi-target and corresponding single-target compounds.

## 2. Materials and Methods 

### 2.1. Screening Compounds with Single- and Multi-Target Activity 

Compounds with single- or multi-target activity were extracted from PubChem BioAssay [24] using in-house scripts supported by Open Eye chemistry toolkits [25]. Initially, all assays with designated targets having PubChem Gene Identifiers were downloaded. Assays against anti-targets or others with reported inconsistent results or designated cytotoxic compounds were omitted. In addition, assays with unusually high hit rates above 2% were removed, which excluded a number of confirmatory assays and very small screens with only few tested compounds from further consideration. Furthermore, assays originating from other databases, such as ChEMBL, were disregarded.

Importantly, for the assessment of compounds with multi-target activity, potentially false positive activity annotations should be avoided as much as possible. To exclude questionable activity annotations, screening compounds were disregarded that yielded one or more alerts in computational filters for pan assay interference compounds (PAINS) [26] from RDKit [27], chemical liable compounds on the basis of empirical medicinal chemistry rules [28], and colloidal aggregators [29]. In addition, confirmed inhibitors of the firefly luciferase (FLuc) enzyme were removed, which are also prone to causing artifacts in some screening assays [30]. Table 1 reports the number of detected compounds with potential interference character. From the original pool of 1,455,765 screening compounds, 151,387 were removed, yielding a final selection of 1,304,387 unique compounds.

The initially obtained set of PubChem assays contained both biochemical and cell-based assay formats and was therefore termed the “mixed” set. From this set, a subset exclusively comprising biochemical single-protein assays was extracted and (termed “biochemical”). Assay set statistics are provided in Table 2. Mixed assays and the biochemical assay subset yielded a total of more than 82 and 28 million compound-target interactions, respectively. 

### 2.2. Datasets for Machine Learning

For both mixed and biochemical assays, compound promiscuity degrees (PDs) were calculated, which were defined as the number of targets a compound was active against. Two different PD thresholds were applied for defining multi-target compounds (MT-CPDs) including PD ≥ 5 (mixed and biochemical assays) and PD ≥ 3 (biochemical assays). For each MT-CPD, a group of corresponding single-target compounds (ST-CPDs) was collected from the initial pool of all compounds (for an ST-CPD, PD = 1). For each target of an MT-CPD, a ST-CPD active against this target, but reported to be inactive against the other targets of the MT-CPD, was randomly selected, if available. Following this procedure, the maximal number of ST-CPDs corresponding to each MT-CPD was determined. For only very few MT-CPDs no corresponding ST-CPDs were identified (and these MT-CPDs were thus omitted). For most MT-CPDs, experimentally confirmed ST-CPDs were not sampled for all targets. Importantly, for each assembled MT-/ST-CPD group, all compounds were tested against all targets, thus ensuring data completeness. Table 3 summarizes the composition of the datasets for ML derived from mixed and biochemical assays, respectively. 

### 2.3. Similarity Calculations

For compound comparison, Tanimoto similarity [31] was calculated on the basis of the Morgan fingerprint with a radius of 2 (folded into 2048-bit vector) [32] from RDKit. This fingerprint systematically captures molecule-specific atom environments for a given bond radius. Distributions of NN similarity values were represented in two-dimensional kernel density estimation (KDE) plots [33].

### 2.4. Machine Learning 

As a molecular representation, the Morgan fingerprint with radius of 2 from RDKit was calculated for each compound. As a control, the standard MACCS structural key (fragment) fingerprint [34] was used as an alternative molecular representation. With respect to machine learning methods, the random forest (RF) [35] and support vector machine (SVM) [36] algorithms were applied. In addition, a k-NN classifier (KNN) was generated. Therefore, training compounds were ranked based upon decreasing fingerprint distances and the majority class label among the k top-ranked compounds was assigned to the test compound. The scikit-learn [37] implementation was used with default parameter settings (version 0.23.2), except for the number of NNs for which optimal *k* values were selected from k ∈ [1,3,5].

RF is a supervised ML algorithm generating an ensemble of independently derived decision trees using bootstrapping for random training data selection. The majority vote of all decision trees determines the predicted class label for test compounds. The scikit-learn RF implementation was used with default hyper-parameter settings (version 0.23.2), except for the number of decision trees (“n_estimators”: 10, 100, 250, 500) and the minimum number of samples required to split an internal node (“min_samples_split”: 2, 3, 5, 7, 10), which were optimized during training.

SVM is another supervised ML algorithm that constructs a hyperplane H in chemical feature space to best separate training instances with different class labels maximizing the margin between them. If linear separation is not possible in a given feature space, projection into higher-dimensional feature space is carried out where linear separation might be feasible. The projection is generated without explicit mathematical mapping through using kernel functions. The relationship between training errors and margin size is controlled by the regularization hyper-parameter C, which was optimized using values of 0.1, 1, 10, 50, 100, 200, 400, 500, 750, and 1000. SVM classifiers were generated with scikit-learn using the linear or Tanimoto kernel [38]. During hyper-parameter optimization, the best performing kernel was selected. 

Test calculations using the alternative fingerprint representations produced similar results and consistently the same trends. Therefore, results obtained with the Morgan fingerprint are presented below.

### 2.5. Calculation Protocols and Performance Measures

A standard double cross validation procedure was applied for each prediction task. In each of the 10 independent external cross validation trials, ST-CPDs of an individual MT-/ST-CPD group were first randomly sampled to balance the dataset and the resulting MT-/ST-CPD pairs were then randomly divided into 80% training and 20% test (sub)sets. For hyper-parameter optimization, MT-/ST-CPD pairs from training sets were randomly divided into 80% and 20% subsets for 10 independent internal cross validation trials. The best performing model was then used to predict test set compounds for the respective external cross validation trial. In addition, the same cross validation procedure was carried out with 50% training and 50% test set data splits, which produced very similar results, with deviations mostly within one standard deviation and consistently low variability of individual trials. Thus, the results were stable for these different test set sizes. 

Five performance measures were applied including balanced accuracy (BA, Equation (1)) [39], Matthew’s correlation coefficient (MCC, Equation (2)) [40], F1 score (Equation (3)) [41], precision (Equation (4)), and recall (Equation (5)).

(1) $$\mathrm{BA}=\frac{1}{2}\left(\mathrm{TPR}+\mathrm{TNR}\right)$$

(2) $$\mathrm{MCC}=\frac{\mathrm{TP}\times \mathrm{TN}-\mathrm{FP}\times \mathrm{FN}}{\sqrt{\left(\mathrm{TP}+\mathrm{FP}\right)\left(\mathrm{TP}+\mathrm{FN}\right)\left(\mathrm{TN}+\mathrm{FP}\right)\left(\mathrm{TN}+\mathrm{FN}\right)}}$$

(3) $$F1=\;2\times \frac{\mathrm{TP}}{2\;\mathrm{TP}+\mathrm{FP}+\mathrm{FN}}$$

TP, TN, FP, and FN stand for true positives, true negatives, false positives, and false negatives, respectively.

Precision reports the proportion of TP among all positive predictions, while recall reports the proportion of recovered TP relative to all positive instances in the dataset:

(4) $$\mathrm{Precision}=\frac{\mathrm{TP}}{\mathrm{TP}+\mathrm{FP}}$$

(5) $$\mathrm{Recall}=\frac{\mathrm{TP}}{\mathrm{TP}+\mathrm{FN}}$$

In addition, predictions were assessed using receiver operating characteristic (ROC) curves [42], which compare the TP rate ([0,1], *y*-axis) to the FP rate ([0,1], *x*-axis) at different classification thresholds. The diagonal corresponds to random class label prediction and an area under the ROC curve (AUC) value of 0.5. Increasing AUC values > 0.50 indicate increasing prediction accuracy, AUC = 1.0 resulting from a perfect prediction.

### 2.6. Structural Relationships

Close structural relationships between screening compounds were detected by applying a molecular fragmentation-based algorithm for the systematic extraction of analog series from compound datasets [43]. Using this approach, the proportion of compounds forming analog relationships with one or more others was determined as a measure of structural diversity. 

## 3. Results

### 3.1. Screening Compounds with Single- and Multi-Target Activity

The data curation procedures described above led to the elimination of ~10% of the pre-selected compounds that might potentially cause assay artifacts (Table 1). The biochemical assay subset comprised ~1/3 of the assays, targets, compounds, and interactions contained in the mixed set (Table 2). A total of 2858 and 1242 MT-CPDs with PD ≥ 5 together with 15,839 and 6629 corresponding ST-CPDs were sampled for ML from mixed and biochemical assays, respectively (Table 3). Applying the lower threshold PD ≥ 3 for MT-CPDs led to the identification of 3468 and 11,793 MT- and corresponding ST-CPDs from biochemical assays and hence further increased the size of the dataset. 

### 3.2. Test Frequencies

**Figure 1:**
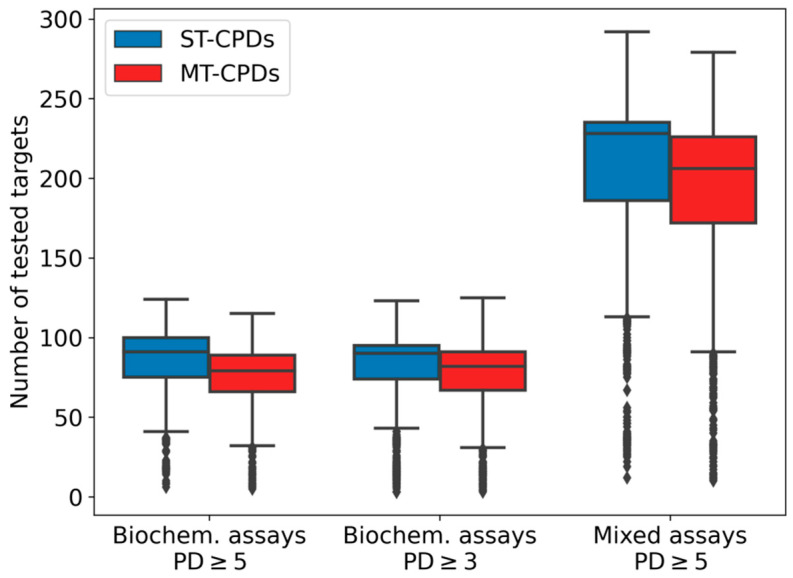
Test frequency of selected compounds. Box plots report the distributions of target-based compound test frequencies for single-target (ST, blue) and multi-target (MT, red) compounds from different datasets. Box plots show the smallest value (lower whisker), lower quartile (lower boundary of the box), median (horizontal line in box), upper quartile (upper boundary of the box), and the maximum value (upper whisker). Values classified as statistical outliers are represented as diamonds.

Prior to balancing of these datasets for ML calculations, we examined the test frequencies of the sampled compounds. The distributions are shown in Figure 1. MT- and ST-CPDs from biochemical assays had comparable test frequencies for both PD thresholds with a median value of ~90 assays per compound. Median test frequency values were slightly higher for ST-CPDs (91 for PD ≥ 5 and 90 for PD ≥ 3) than for MT-CPDs (79 for PD ≥ 5 and 82 for PD ≥ 3). Compounds from mixed assays were evaluated in even larger number of assays, with a median test frequency of 228 and 206 assays per ST- and MT-CPD, respectively. Hence, selected compounds were extensively tested, with comparably large numbers of assays for ST- and MT-CPDs. Accordingly, these datasets provided a sound basis for sampling of compounds to balance training and test sets for ML analysis.

### 3.3. Varying Conditions for Machine Learning 

ML calculations were carried out under three different conditions. First, compounds from original (“full”) datasets were used. Second, 50% of MT-/ST-CPD groups were randomly removed (“random removal”). These calculations were carried out to examine the influence of dataset size reduction on prediction accuracy and served as a control for the third condition. In the third case, 25% of MT-/ST-CPD groups with ST-CPDs most similar to other ST-CPDs and 25% of groups with MT-CPDs most similar to other MT-CPDs were discarded (“NN removal”), which also resulted in a 50% net reduction in dataset size. Hence, NN relationships of both MT- and ST-CPDs were detected using calculated Tanimoto similarity values and systematically reduced.

### 3.4. Nearest Neighbor Relationships

**Figure 2:**
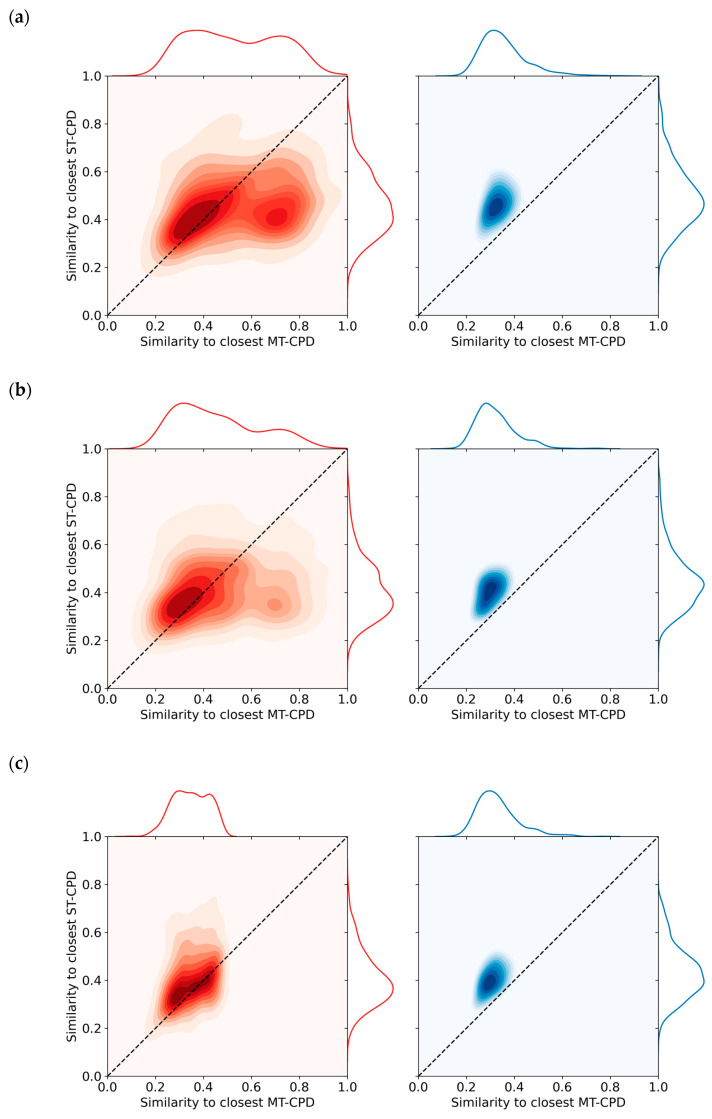
Nearest neighbor relationships for single- and multi-target compounds from biochemical assays. KDE plots report the distribution of closest similarity relationships. For each MT- and ST-CPD, all pairwise Tanimoto similarities were calculated. Similarity values for the NNs of all MT- (x-axis) and ST-CPDs (y-axis) are reported. The joint distribution is displayed as a KDE plot for MT-CPDs (red, left) and ST-CPDs (blue, right). In KDE plots, color intensity corresponds to different regions of data density, as delineated with contour lines. In addition, the underlying similarity value distributions are plotted on the top and right scale, respectively. Compounds falling onto the diagonal have the same closest similarity value to ST- and MT-CPDs. In (**a**), the similarity value distributions are reported for the original data set. In (**b**) and (**c**), NN calculations according to (**a**) were carried out after random removal of 50% of the MT- and ST-CPDs and removal of 50% of the NNs from MT- and ST-CPDs, respectively.

**Figure 3:**
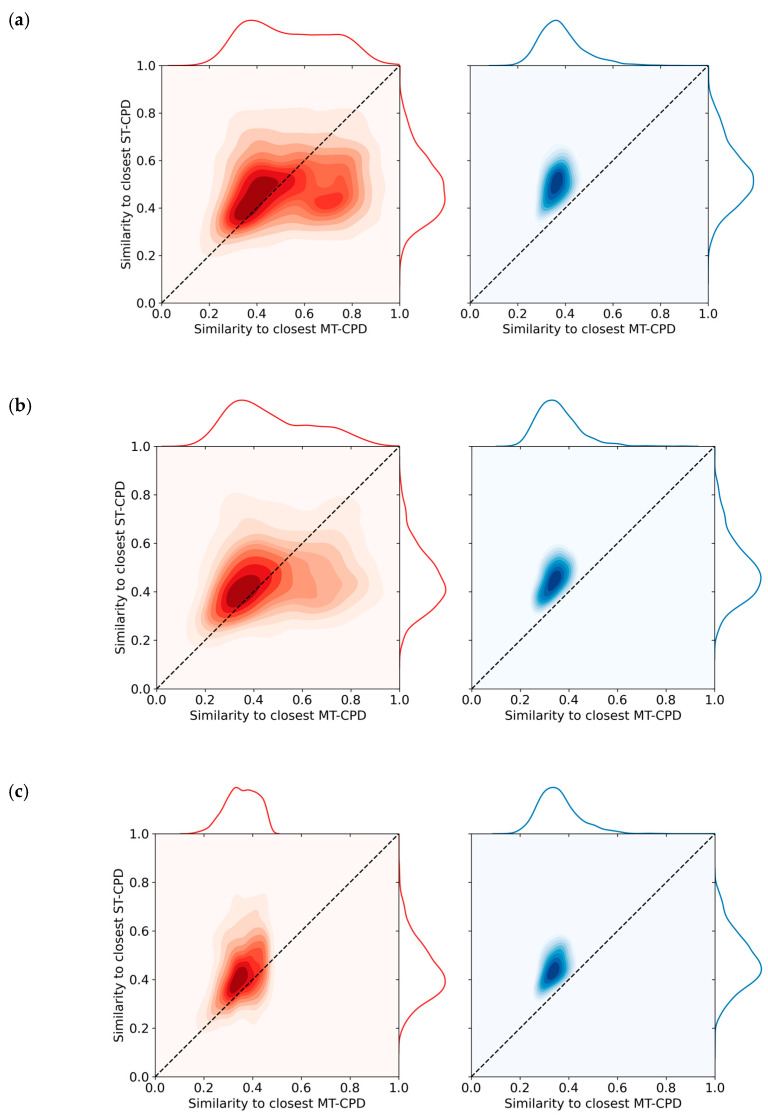
Nearest neighbor relationships for single- and multi-target compounds from mixed assays. KDE plots report the distribution of closest similarity relationships according to Figure 2. In (**a**), the similarity value distributions are reported for the original data set. In (**b**,**c**), NN calculations were carried out after random removal of 50% of the MT- and ST-CPDs and removal of 50% of the NNs from MT- and ST-CPDs, respectively.

Figure 2 shows the distribution of closest Tanimoto similarity values for MT- and ST-CPD from biochemical assays in original and reduced datasets. The distribution for the original dataset in Figure 2a reveals a general tendency for MT-CPDs to be more similar to another MT-CPD than to ST-CPDs. For ST-CPDs, a similar but weaker tendency was detected because the densely populated part of the distribution mapped closely to the diagonal in the plot. Random compound removal did not notably change the NN similarity value distributions, as shown in Figure 2b. Accordingly, there was no significant effect of random compound removal on the distribution of NN relationships. By contrast, as shown in Figure 2c, equally sized removal of NN instances substantially altered the distribution for MT-CPDs (but not ST-CPDs) such that the densely populated part of the distribution mapped to the diagonal. In addition, many MT-CPDs became most similar to an ST-CPD after removal of their original NNs, which were predominantly MT-CPDs. Equivalent observations were made for MT- and ST-CPDs from mixed assays, as shown in Figure 3a for the original dataset and in Figure 3b,c for the reduced datasets following random compound or NN removal, respectively.

### 3.5. Analog Relationships

The analysis of NN relationships among MT- and corresponding ST-CPDs was complemented by the systematic detection of structural analogs (sharing the same core structure) in the different datasets. For compounds from biochemical assays applying the PD ≥ 5 threshold for MT-CPDs, 59% of the MT- and 40% of the ST-CPDs were found to have structural analogs. When the PD ≥ 3 threshold was applied the proportions slightly increased to 64% of the MT- and 52% of the ST-CPDs. For compounds from mixed assays applying the PD ≥ 5 threshold, structural analogs were identified for 62% of the MT- and 56% of the ST-CPDs. Thus, analog relationships were similarly distributed in the datasets. However, structural analogs were detected for ~10–20% more MT- than ST-CPDs, consistent with the stronger tendency of MT-CPDs to form NN relationships with other MT-CPDs than observed for ST-CPDs, as discussed above. Notably, for approximate half of the compounds from all sets, no structural analogs were detected, indicating that the compounds were overall structurally diverse, as typically expected for collections of biological screening compounds.

### 3.6. Diagnostic Machine Learning 

Diagnostic ML models were built on the basis of chemical structure to systematically distinguish between MT- and ST-CPDs under different conditions, as detailed above. The principal idea behind diagnostic ML was to determine prediction accuracy as an indicator of the presence of structural features that systematically differentiate between promiscuous and non-promiscuous compounds. If such features or patterns do not exist, ML models based upon chemical structure could not possibly be predictive. Thus, diagnostic ML did not aim to optimize model performance but associate observed prediction accuracy with varying calculation conditions to determine the presence or absence of features that might differentiate between promiscuous and non-promiscuous compounds. Given our system setup, removal of NNs from datasets systematically reduced close structural relationships and therefore served as an important control.

### 3.7. Target Coverage

**Figure 4:**
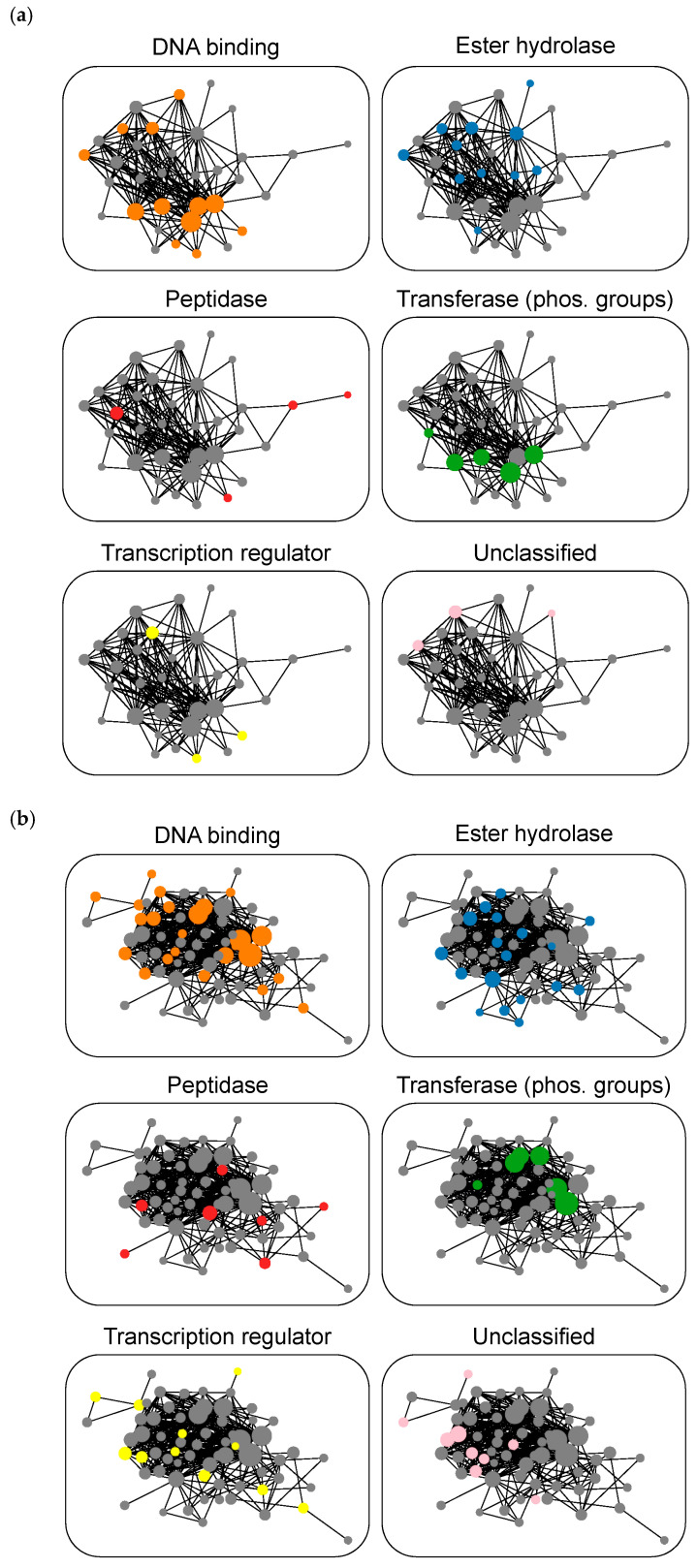
Target networks for multi-target compounds. In (**a**,**b**), networks are shown for MT-CPDs from biochemical and mixed assays, respectively. Each node represents an individual target that is scaled in size according to the number of its MT-CPDs. Nodes are connected by edges if the corresponding targets share at least 50 MT-CPDs. Since a target may have multiple functional annotations, all panels display the same target network colored according to the absence (gray) or presence of a given protein function (orange, DNA binding; blue, ester hydrolase; red, peptidase; green, phosphorous group transferase; yellow, transcription regulator; pink, unclassified).

We also determined the target coverage of MT-CPDs extracted from biochemical and mixed assays. Therefore, target annotations of MT-CPDs were recorded and compound-based target networks were generated. In these networks, targets were represented as nodes, which were connected by an edge if they shared a pre-defined number of MT-CPDs. Figure 4a,b show target network representations for MT-CPDs from biochemical and mixed assays, respectively. In these cases, targets were connected if they shared at least 50 MT-CPDs. In both instances, the major network component was a densely connected cluster of nearly the same target composition. This central network component remained stable when different MT-CPD thresholds were evaluated. Accordingly, mixed assays and their biochemical assay subset defined essentially the same target space predominantly (but not exclusively) consisting of DNA binding proteins including transcription regulators, peptidase and ester hydrolase enzymes, phospho-transferases, and small numbers of unclassified targets. Hence, compared to MT-CPDs compounds from medicinal chemistry sources [23], target space covered by MT-CPDs and corresponding ST-CPDs from biological screens was more confined. While MT-CPDs from biochemical and mixed assays defined a very similar target space, target coverage further increased for the larger set of mixed assays, as also illustrated by increasing network density for MT-CPDs from mixed compared to biochemical assays (Figure 4).

### 3.8. Predictions for Compounds from Biochemical Assays 

**Figure 5:**
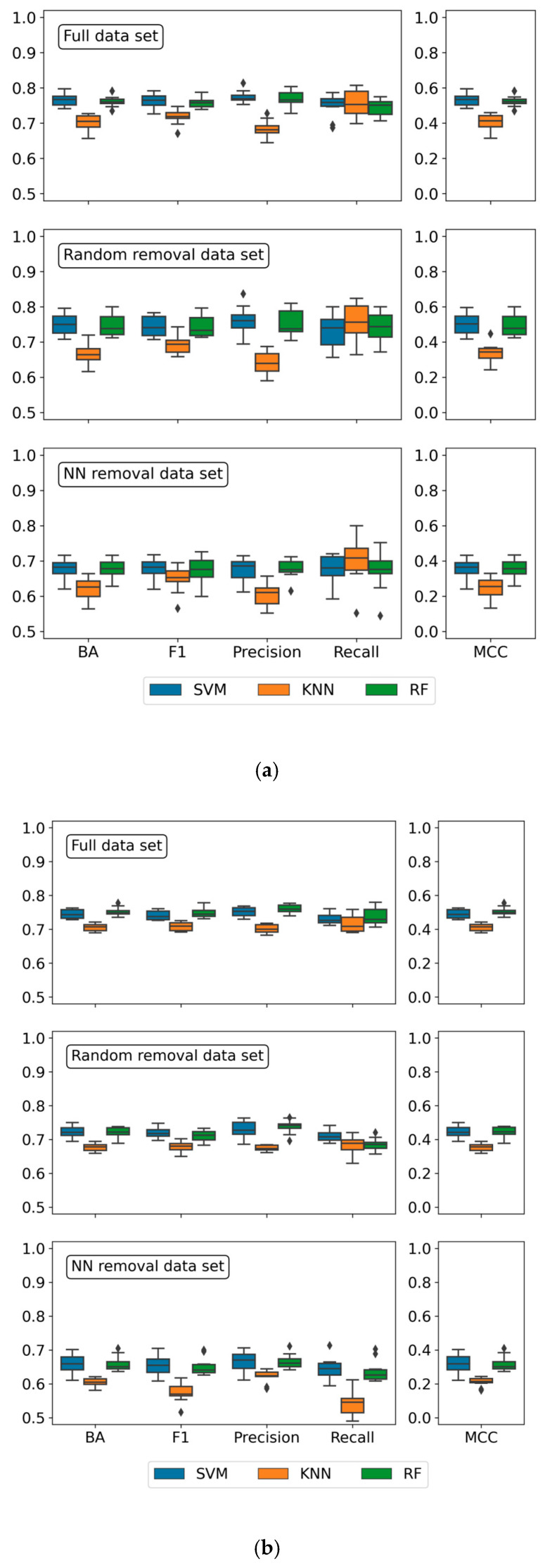
Comparison of prediction accuracies. In (**a**,**b**), prediction accuracies are shown for data sets from biochemical and mixed assays, respectively. Box plots report the distribution of balanced accuracy values for 10 independent SVM (blue), KNN (orange), and RF (green) trials using different training and test sets. From the top to the bottom, results are shown for all ST- and MT-CPDs (All CPDs), reduced data sets after random removal of 50% of the compounds (Random removal), and corresponding data sets after removal of 50% of NNs (NN removal).

**Figure 6:**
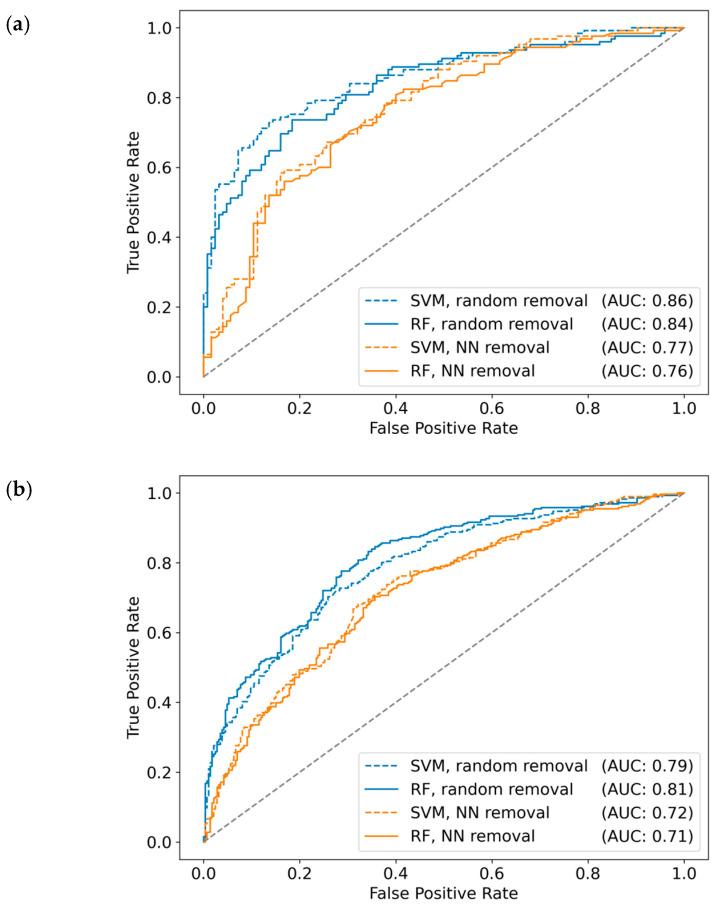
Receiver operating characteristic curves. For SVM and RF classification of ST- vs. MT-CPDs with random compound or NN removal, ROC curves report prediction results for the first compound data split. In (**a**,**b**), results are shown for data sets from biochemical and mixed assays, respectively.3.9. Predictions for Compounds from Mixed Assays

ML models were first generated for MT- and ST-CPDs from biochemical assays, which represented single-target assays probing direct compound-target interactions. Initially, datasets obtained for the PD ≥ 5 threshold were used. Results of the predictions are reported in Table 4 and Figure 5a. The models were generally found to be predictive, reaching an accuracy of up to 80%, as assessed on the basis of alternative performance measures. Furthermore, model performance was generally stable across different cross validation trials, as indicated by narrow value distributions captured in box plots (Figure 5a). For the full datasets, only relatively minor differences in performance were observed for RF and SVM models. KNN models were less predictive but still yielded a median accuracy above 70%, thus indicating an important role of NN effects for predictions. The largest differences between RF/SVM and KNN models were observed on the basis of MCC values (0.41 for KNN, 0.52 for RF, 0.53 for SVM) and precision (0.68 for KNN, 0.77 for RF and SVM). Random removal of MT- and ST-CPDs consistently decreased the performance for all three model types on the basis of all measures, albeit by only small margins (within a range of 0.01 to 0.08, depending on the measure). This small decrease in performance was attributable to the reduction in available training data. However, NN-based compound removal of the same magnitude as random removal consistently resulted in a larger decrease in model performance, especially for KNN classifiers. While the ML models remained predictive, accuracy was generally lower than 70%. Figure 6a compares ROC curves for predictions of reduced datasets. After random compound removal, SVM and RF models still yielded AUC values of 0.86 and 0.84, respectively, whereas NN removal reduced these values to 0.77 (SVM) and 0.76 (RF). Taken together, predictions on reduced datasets confirmed a strong influence of NN effects on model accuracy.

To examine the potential influence of the PD threshold for defining MT-CPDs, the analysis was then repeated for datasets resulting from PD ≥ 3. The same trends were observed but prediction accuracy was slightly lower than for the PD ≥ 5 datasets, consistent with the assumption that increasingly promiscuous MT-CPDs should be easier to distinguish from ST-CPDs. Results obtained for the PD ≥ 3 datasets are provided as Supplementary Materials.

In light of the findings discussed above, we further extended diagnostic ML analysis to compounds from mixed assays applying the PD ≥ 5 threshold. These assays included different formats such as biochemical and cell-based assays, which increased assay heterogeneity and, thus, decreased the confidence level of target annotations compared to biochemical assays. On the other hand, dataset size and target coverage by mixed assays further increased compared to the biochemical assay subset. 

Results of the predictions on datasets from mixed assays are reported in Table 5 and Figure 5b. Reassuringly, the results essentially displayed the same trends as discussed above. Overall, prediction accuracy was slightly reduced for the more heterogeneous mixed datasets, but RF and SVM models also approached 80% prediction accuracy on the full set. These models performed better than KNN classifiers, which were also predictive in this case. The largest difference was again observed on the basis of MCC values (0.41 for KNN, 0.51 for RF, 0.49 for SVM). Control calculations with the randomly reduced sets displayed only a minor reduction in model performance, as observed before. By contrast, following NN-based compound removal, the relative reduction in performance was larger than determined for compounds from biochemical assays, especially for KNN classification where MCC values were reduced from 0.41 (full set) to 0.24. Figure 6b compares ROC curves for predictions of reduced datasets from mixed assays. In this case, random compound removal yielded AUC values of 0.79 (SVM) and 0.81 (RF) that were reduced by NN removal to 0.72 and 0.71, respectively. Taken together, these findings also revealed a marked influence of NN effects on the predictions, as observed for datasets from biochemical assay.

However, ML models derived from all datasets were predictive, strongly indicating the presence of structural features distinguishing between MT- and corresponding ST-CPDs. 

## 4. Discussion

If test frequencies of compounds are unknown, which is typically the case for compounds reported in the medicinal chemistry literature, promiscuity analysis generally includes a factor of uncertainty, due to data sparseness. Accordingly, PD values might be under-estimated and calculated differences in promiscuity remain uncertain. As long as the ultimate goal of chemogenomics efforts is not reached (i.e., testing “all” compounds against “all” targets), data incompleteness will continue to play a critical role in the assessment of target specificity vs. multi-target activity, which is of equal relevance for chemical biology and drug discovery. By focusing promiscuity analysis on large volumes of screening data, the data incompleteness issue can be addressed by taking test frequencies and negative assay results directly into account, as demonstrated herein. Compared to exploring multi-target activities in the very large space of currently more than 10,000 targets for which medicinal chemistry has identified active compounds, focusing the analysis on screening data also comes at a price. Target space defined by screening campaigns is typically smaller and, in addition, there are uncertainties associated with activity measurements, due to screening assay noise. Hence, from this point of view, promiscuity analysis of compounds from medicinal chemistry and screening sources are complementary in nature. 

Further increasing the level of confidence for the assignment of single- vs. multi-target activity for promiscuity analysis via diagnostic ML has been a prime motivation for our current study. Therefore, we have devised a data curation protocol focusing the analysis on compounds from different types of assays that were extensively tested in comparably large numbers of assays. Furthermore, by equally taking positive and negative assay readouts into account, groups of MT- and corresponding ST-CPDs were assembled ensuring data completeness in each case. Moreover, care was taken to remove all screening compounds with potential assay liabilities from further consideration, hence reducing the likelihood of including false positive active compounds in the analysis as much as possible. Datasets from biochemical and mixed assays were assembled under stringent selection criteria and then further modified on the basis of NN analysis to assess the influence of NN relationships between active compounds on predictions. The influence of dataset size reduction was determined by generating equally sized reduced datasets through random compound or NN removal, lending further credence to the analysis.

Given the premise of diagnostic ML, models were built on the basis of chemical structure to systematically distinguish between MT- and corresponding ST-CPDs under varying conditions using specifically modified datasets. Notably, for such diagnostic ML analysis, there is no external or experimental validation scheme that might be applicable. The ML calculations were complemented by NN analysis and revealed that diagnostic models were generally predictive and that NN relationships between MT- and ST-CPDs had a strong influence on the predictions. 

On the basis of our findings, the picture is emerging that MT- and ST-CPDs have distinguishing structural features that can be detected via ML. These features can at least in part be associated with NN relationships. In other words, many MT-CPDs are more similar to other MT-CPDs than corresponding ST-CPDs and vice versa. Investigating whether these structural relationships might depend on the presence or absence of general structural signatures of compound promiscuity or of structural features that are confined to given compound classes and target combinations provides interesting opportunities for future research resulting from our analysis.

## 5. Conclusions

In this work, we have systematically assessed the ability of ML to distinguish between promiscuous and non-promiscuous compounds from biological screening assays explicitly taking test frequencies and positive, as well as negative assay readouts, into account. Diagnostic ML models based upon chemical structure were consistently able to distinguish between MT- and corresponding ST-CPDs, providing evidence for the presence of differentiating structural features, and a strong positive influence of NN relationships on prediction accuracy was detected. MT-CPDs were often more similar to each other than to corresponding ST-CPDs, and the same applied to ST-CPDs, albeit to a lesser extent. These relationships supported the predictions. The results reported herein were encouraging from two points of view. First, predictive performance and equivalent trends were consistently observed under different calculation conditions and for compounds from biochemical assays and more heterogeneous mixed assays. Second, the observed trends essentially paralleled earlier findings from complementary studies on compounds from medicinal chemistry sources. Thus, taken together, these investigations assign a high level of confidence to the conceptual framework of promiscuity analysis reported herein. Our predictions demonstrated that MT-CPDs from screening sources, which were not further optimized for known activities, could generally be predicted with reasonable to high accuracy, hence providing a basis for practical applications in chemical biology and drug discovery.

## Figures and Tables

**Table 1:** Compound removal during data curation.

Curation Criteria	Number of Compounds
Original pool	1,455,765
Potential PAINS	79,563
Aggregators	11,279
Potential chemical liabilities	21,214
FLuc inhibitors	27,653
Cytotoxic compounds	24,323
Final selection	1,304,387

**Table 2:** Statistics for mixed and biochemical assay sets.

Number of	Mixed Assay Set	Biochemical Assay Subset
Assays	817	259
Targets	398	143
Compounds	1,304,108	493,229
Interactions	82,807,570	28,345,295

**Table 3:** Composition of datasets for machine learning.

Dataset	Mixed	Biochemical	
	PD ≥ 5	PD ≥ 5	PD ≥ 3
MT-CPDs	2858	1242	3468
ST-CPDs	15,839	6629	11,793

**Table 4:** Prediction accuracy for single- versus multi-target compounds from biochemical assays.

Set	Algorithm	BA	F1	MCC	Precision	Recall
Full set	KNN	0.70 ± 0.03	0.72 ± 0.03	0.41 ± 0.05	0.68 ± 0.03	0.76 ± 0.04
	RF	0.76 ± 0.02	0.76 ± 0.02	0.52 ± 0.04	0.77 ± 0.03	0.75 ± 0.03
	SVM	0.77 ± 0.02	0.76 ± 0.02	0.53 ± 0.04	0.77 ± 0.02	0.75 ± 0.04
Random Removal	KNN	0.66 ± 0.04	0.69 ± 0.04	0.33 ± 0.07	0.64 ± 0.04	0.76 ± 0.06
	RF	0.75 ± 0.04	0.75 ± 0.04	0.50 ± 0.07	0.75 ± 0.04	0.74 ± 0.05
	SVM	0.75 ± 0.03	0.74 ± 0.03	0.50 ± 0.06	0.76 ± 0.04	0.73 ± 0.05
NN Removal	KNN	0.62 ± 0.04	0.65 ± 0.04	0.24 ± 0.07	0.60 ± 0.04	0.70 ± 0.08
	RF	0.68 ± 0.03	0.67 ± 0.03	0.35 ± 0.06	0.68 ± 0.03	0.67 ± 0.07
	SVM	0.68 ± 0.04	0.68 ± 0.04	0.35 ± 0.07	0.68 ± 0.04	0.68 ± 0.05

**Table 5:** Prediction accuracy for single- versus multi-target compounds from mixed assays.

Set	Algorithm	BA	F1	MCC	Precision	Recall
Full set	KNN	0.71 ± 0.02	0.71± 0.02	0.41 ± 0.03	0.70 ± 0.02	0.72 ± 0.03
	RF	0.75 ± 0.02	0.75± 0.02	0.51 ± 0.03	0.76 ± 0.02	0.74 ± 0.03
	SVM	0.74 ± 0.02	0.74± 0.02	0.49 ± 0.03	0.75 ± 0.02	0.73 ± 0.02
Random Removal	KNN	0.68 ± 0.02	0.68± 0.02	0.35 ± 0.03	0.67 ± 0.01	0.68 ± 0.03
	RF	0.72 ± 0.02	0.71± 0.02	0.44 ± 0.04	0.74 ± 0.03	0.69 ± 0.02
	SVM	0.72 ± 0.02	0.72± 0.02	0.45 ± 0.04	0.73 ± 0.03	0.71 ± 0.02
NN Removal	KNN	0.60 ± 0.02	0.57± 0.03	0.21 ± 0.03	0.62 ± 0.02	0.54 ± 0.06
	RF	0.66 ± 0.03	0.65± 0.03	0.32 ± 0.05	0.67 ± 0.03	0.64 ± 0.04
	SVM	0.66 ± 0.03	0.65± 0.03	0.32 ± 0.06	0.67 ± 0.03	0.64 ± 0.04

## Supplementary Materials

The following are available online at https://www.mdpi.com/2218-273X/10/12/1605/s1, Figure S1. Nearest neighbor relationships for single- and multi-target compounds with PD ≥3 from biochemical assays, Figure S2. Target networks for multi-target compounds with PD ≥ 3 from biochemical assays, Figure S3. Comparison of prediction accuracies for classification of single-target vs. multi-target compounds with PD ≥ 3 from biochemical assays, Figure S4. Receiver operating characteristic curves for classification of single-target vs. multi-target compounds with PD ≥ 3 from biochemical assays, Table S1. Prediction accuracy for single- versus multi-target compounds with PD ≥ 3 from biochemical assays.
