# Supplementary material for: Analysis of Biological Screening Compounds with Single- or Multi-Target Activity via Diagnostic Machine Learning
Source: Biomolecules. 2020 Nov 27;10(12):1605. doi: 10.3390/biom10121605 (PMC7761051; doi:10.3390/biom10121605)
Supplement: Supplementary file 1 [file biomolecules-10-01605-s001.pdf]

## Supplementary Information

# Analysis of Biological Screening Compounds with Single- or Multi-Target Activity via Diagnostic Machine Learning

**Christian Feldmann, Dimitar Yonchev and Jürgen Bajorath ***

Department of Life Science Informatics, B-IT, LIMES Program Unit Chemical Biology and Medicinal Chemistry, Rheinische Friedrich-Wilhelms-Universität, Endenicher Allee 19c, D-53115 Bonn, Germany; cfeldmann@bit.uni-bonn.de (C.F.); yonchev@bit.uni-bonn.de (D.Y.)

* Correspondence: bajorath@bit.uni-bonn.de; Tel.: +49-228-73-69100

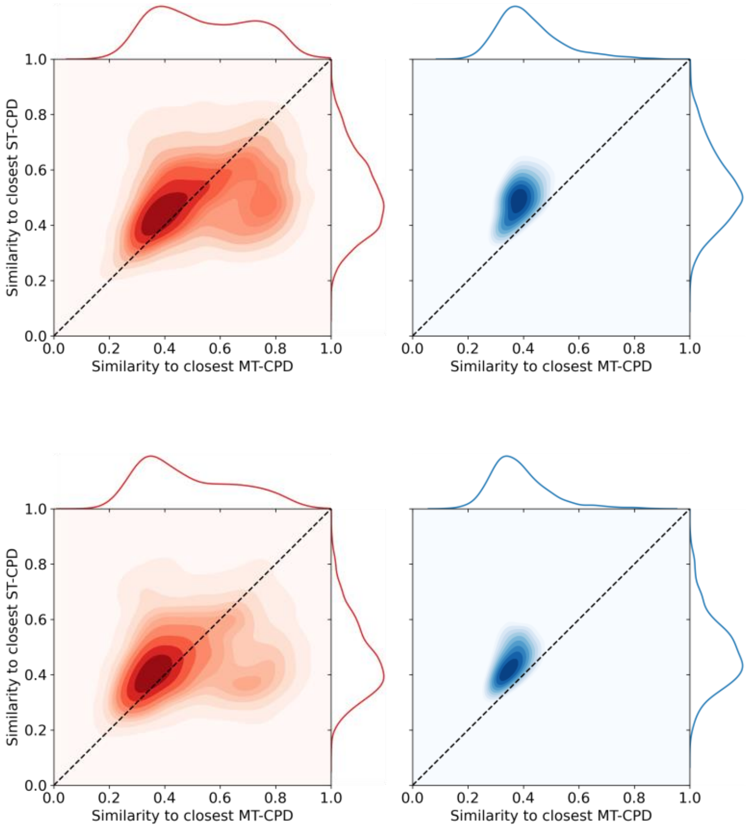

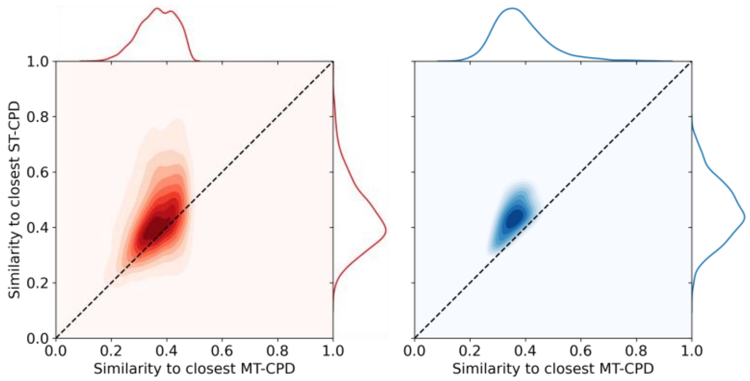


**Figure S1.** Nearest neighbor relationships for single- and multi-target compounds with PD ≥3 from biochemical assays. KDE plots report the two-dimensional distribution of Tanimoto similarities for represented compounds (MT-CPDs: red, ST-CPDs: blue) to most similar MT- (x-axis) and ST-CPDs (y-axis) according to Figure 2.

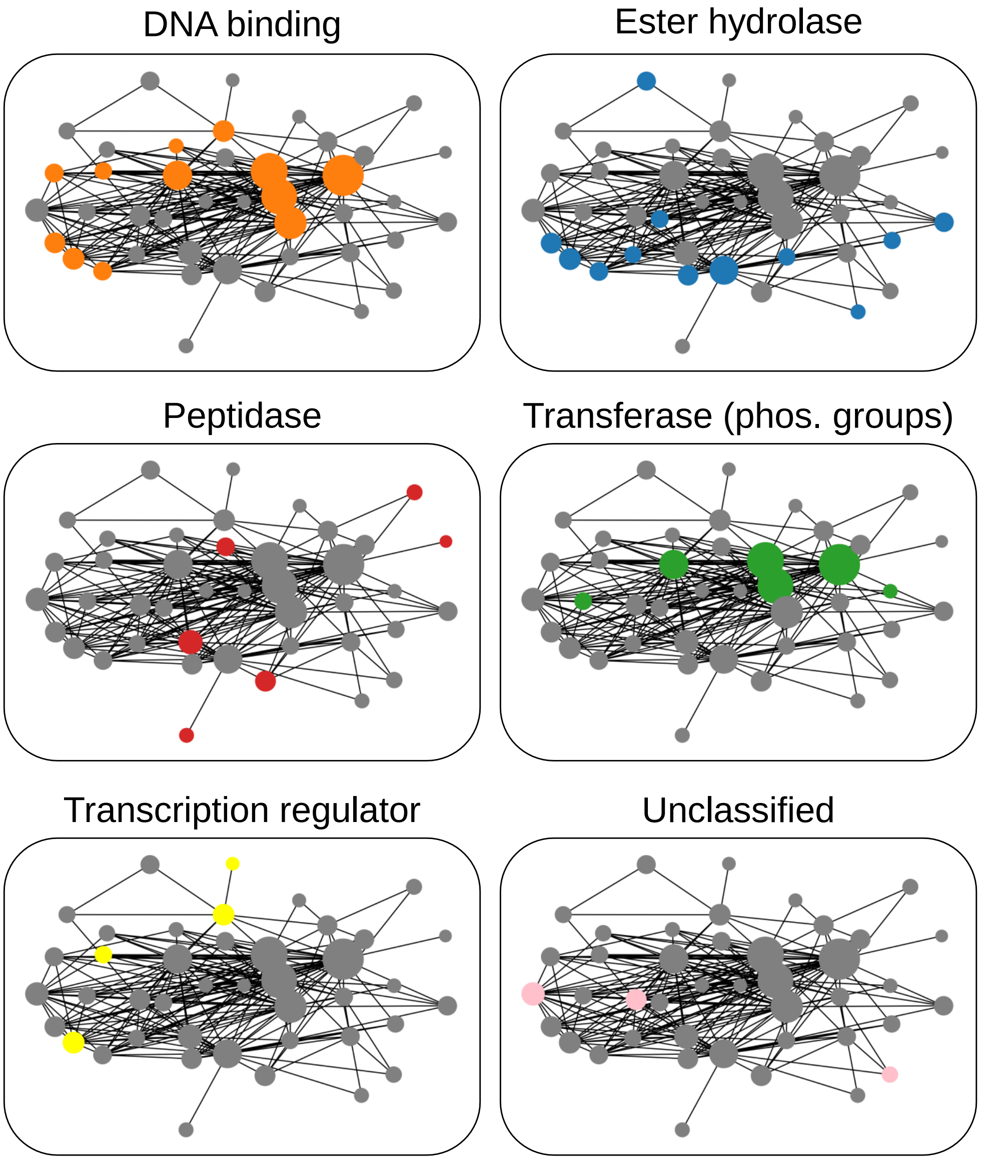


**Figure S2.** Target networks for multi-target compounds with PD ≥ 3 from biochemical assays. The network represents targets as nodes that are connected if they share at least 50 MT-CPDs. The size of each node is scaled according to the total number of MT-CPDs active against the represented target. The network is depicted multiple times with different color annotations based on the presence (orange, DNA binding; blue, ester hydrolase; red, peptidase; green, phosphorous group transferase; yellow, transcription regulator; pink, unclassified) or absence (gray) of a protein function annotation for a given target.

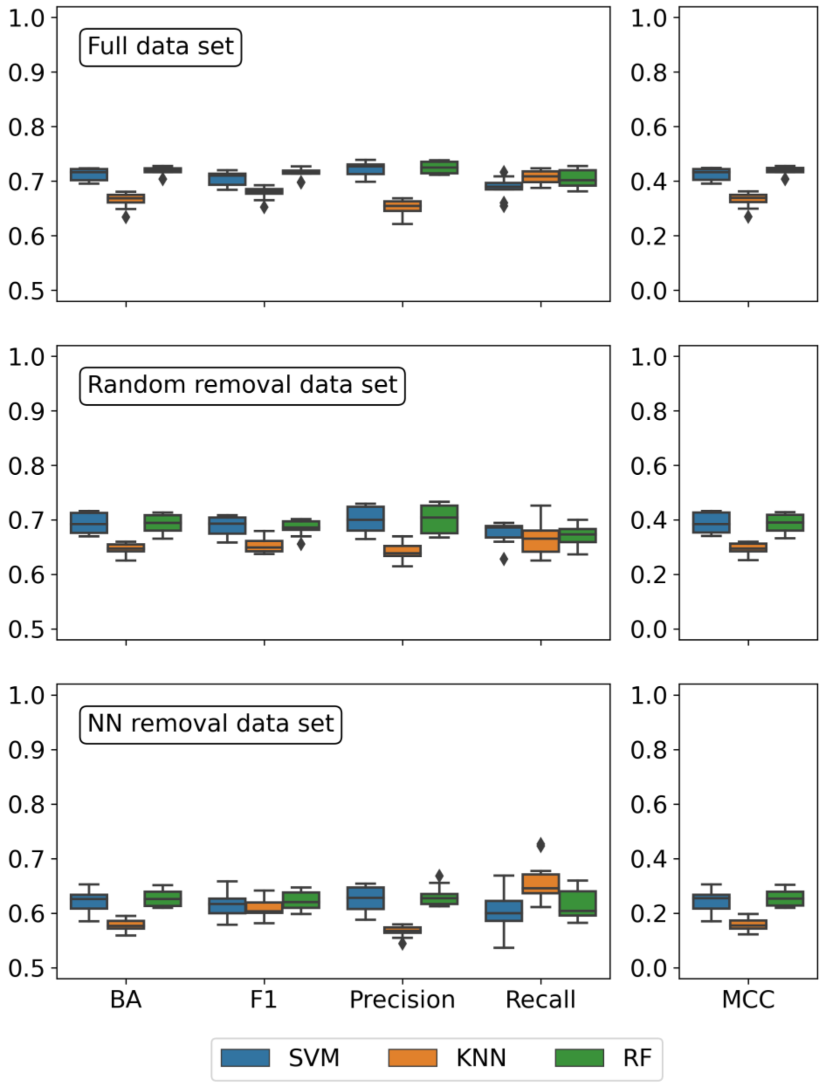


**Figure S3.** Comparison of prediction accuracies for classification of single-target vs. multi-target compounds with PD ≥ 3 from biochemical assays. Box plots represent performance distributions of SVM (blue), KNN (orange), RF (green) for 10 independent trials. Reported metrics are balanced accuracy (BA), F1 score (F1), precision, recall, and Matthew's correlation coefficient (MCC). Results are listed for the full data set (top), 50% random removal data set (center) and 50% NN removal data set (bottom).

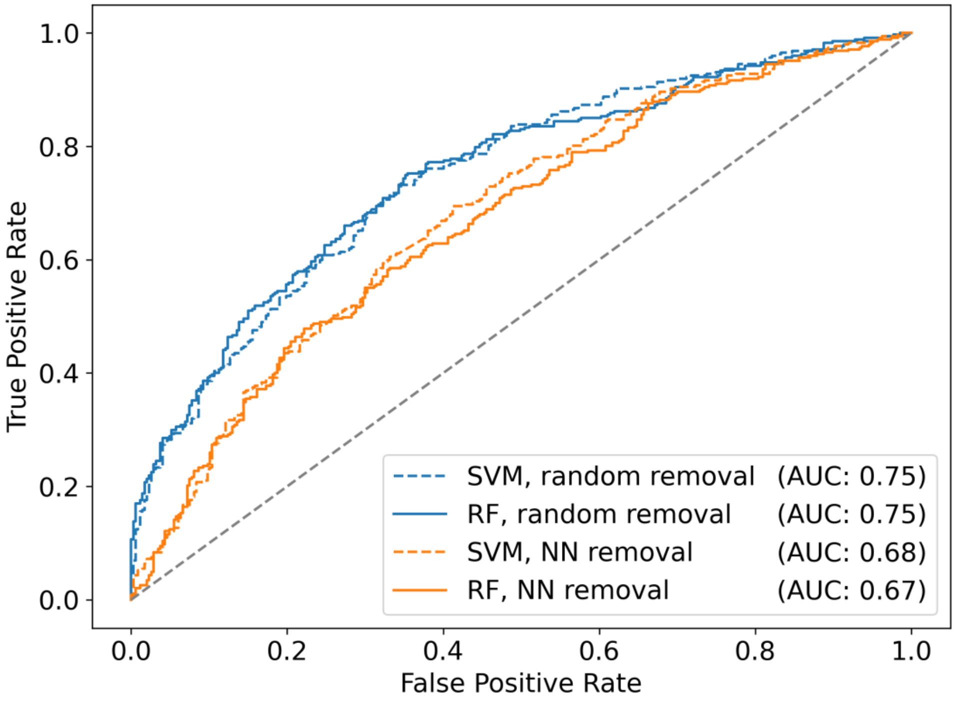


**Figure S4.** Receiver operating characteristic curves for classification of single-target vs. multi-target compounds with PD ≥ 3 from biochemical assays. ROC curves report the performance of SVM and RF models on predicting ST- vs. MT-CPDs at different classification thresholds. Shown are the results for data sets reduced via random and nearest neighbor (NN) compound removal, respectively (analogous to Figure 6).

**Table S1. Prediction accuracy for single- versus multi-target compounds with PD ≥ 3 from biochemical assays.**

| Set | Algorithm | BA | F1 | MCC | Precision | Recall |
|---|---|---|---|---|---|---|
| Full set | KNN | 0.66 ± 0.02 | 0.68 ± 0.02 | 0.33 ± 0.03 | 0.65 ± 0.02 | 0.71 ± 0.02 |
| | RF | 0.72 ± 0.01 | 0.71 ± 0.01 | 0.44 ± 0.02 | 0.72 ± 0.02 | 0.70 ± 0.02 |
| | SVM | 0.71 ± 0.02 | 0.70 ± 0.02 | 0.42 ± 0.03 | 0.72 ± 0.02 | 0.69 ± 0.02 |
| Random Removal | KNN | 0.65 ± 0.02 | 0.65 ± 0.02 | 0.29 ± 0.03 | 0.64 ± 0.02 | 0.67 ± 0.04 |
| | RF | 0.69 ± 0.02 | 0.69 ± 0.02 | 0.38 ± 0.04 | 0.70 ± 0.03 | 0.67 ± 0.02 |
| | SVM | 0.69 ± 0.02 | 0.69 ± 0.02 | 0.39 ± 0.04 | 0.70 ± 0.03 | 0.68 ± 0.03 |
| NN Removal | KNN | 0.58 ± 0.02 | 0.61 ± 0.02 | 0.16 ± 0.03 | 0.57 ± 0.02 | 0.66 ± 0.04 |
| | RF | 0.63 ± 0.02 | 0.62 ± 0.02 | 0.25 ± 0.04 | 0.63 ± 0.02 | 0.61 ± 0.03 |
| | SVM | 0.62 ± 0.02 | 0.61 ± 0.03 | 0.24 ± 0.04 | 0.63 ± 0.03 | 0.60 ± 0.04 |
